# Challenges in Perioperative Cerebral Oximetry Monitoring: A Case of Clofazimine-Induced Skin Pigmentation Interference

**DOI:** 10.7759/cureus.42988

**Published:** 2023-08-05

**Authors:** Rafaela Silva, Joana Veiga, João Barbosa, Celina Oliveira, Filipa Carvalho

**Affiliations:** 1 Anesthesiology Department, Hospital de Braga, Braga, PRT; 2 Anesthesiology Department, Centro Hospitalar de Vila Nova de Gaia e Espinho, Vila Nova de Gaia, PRT

**Keywords:** cerebro vascular disesase, skin pigmentation, cerebral oximetry, neuro-monitoring, monitoring

## Abstract

Perioperative stroke is a potentially devastating complication in patients undergoing noncardiac surgery. The most consistent risk factor associated with the condition is a history of a prior stroke. Cerebral oximetry is a simple, non-invasive, and continuous monitoring device that uses near-infrared spectroscopy (NIRS) to monitor cerebral oxygenation. However, like other monitoring devices, cerebral oximetry has certain limitations, and it must be interpreted cautiously and by taking into account all available clinical information related to the patient. We present a case of a 62-year-old Caucasian woman with a past medical history of a transient ischemic attack (TIA), who had been advised to undergo a right pneumectomy by video-assisted thoracoscopic surgery for treating chronic infection of bronchiectasis. Before administering any drug and while the patient was still alert, we monitored NIRS, and the values recorded were 15 on the left side and 26 on the right side. Despite being Caucasian, she had a darker brownish skin color due to chronic clofazimine use, which is known to cause skin pigmentation. Skin pigmentation is known to attenuate the transmission of near-infrared (NIR) light, potentially affecting the estimation of cerebral oxygen saturation. Thus, our patient suffered from clofazimine-induced skin pigmentation, which may have interfered with the NIR light transmission, which explains the extremely low values observed.

Regional intracerebral oxygen saturation should be interpreted in the context of all available clinical information since NIRS transmission can be influenced by several factors and skin pigment has been found to independently influence regional intracerebral oxygen saturation. Apart from race or high serum bilirubin concentration, we should also consider other causes of skin pigmentation alterations, such as pharmacological therapy.

## Introduction

Perioperative stroke is a potentially severe complication in patients undergoing noncardiac surgery, representing a significant public health burden [1]. The most consistent risk factor is a history of a prior stroke [2]. Patients with a history of transient ischemic attack (TIA) also have a similar risk of perioperative stroke and often have silent infarcts [2]. Exposure to anesthesia and surgery has been found to be an independent risk factor for stroke even after non-high-risk surgeries, probably because they may cause perturbations in cardiac output, cerebral metabolism, and oxygenation leading to the precipitation of perioperative stroke [2]. Therefore, one of the fundamental objectives of the anesthesiologist is the maintenance of adequate oxygen delivery to the brain. However, the brain remains one of the least monitored organs during anesthesia [3]. Cerebral oximetry is a continuous monitoring device that uses near-infrared spectroscopy (NIRS) to monitor cerebral oxygenation. It is a simple, non-invasive monitoring methodology that may improve patient outcomes in a variety of different clinical situations [3]. However, it is associated with certain limitations, such as interference from extracranial blood sources or electrosurgical equipment [3]. Since it is a regional cerebral oxygenation monitor, there are large areas of the brain that remain unmonitored, and it also fails to identify the cause of desaturation [3]. Furthermore, melanin, the predominant skin pigment, is known to attenuate near-infrared (NIR) light transmission, potentially affecting the estimation of cerebral oxygen saturation, as reported in African American [4] and jaundiced patients [5].

Skin hyperpigmentation can be drug-induced in approximately 10-20% of cases and one of the agents associated with it is clofazimine [6]. Clofazimine is an antibiotic used for the treatment of leprosy, mycobacterial infection, or multidrug-resistant tuberculosis. Clofazimine-induced skin pigmentation, its most common side effect, is observed in more than 94% of the patients and usually resolves with the discontinuation of the drug [6]. We present a case in which clofazimine-induced skin pigmentation interfered with cerebral oximetry monitoring, thereby hindering its use.

## Case presentation

**Figure 1:**
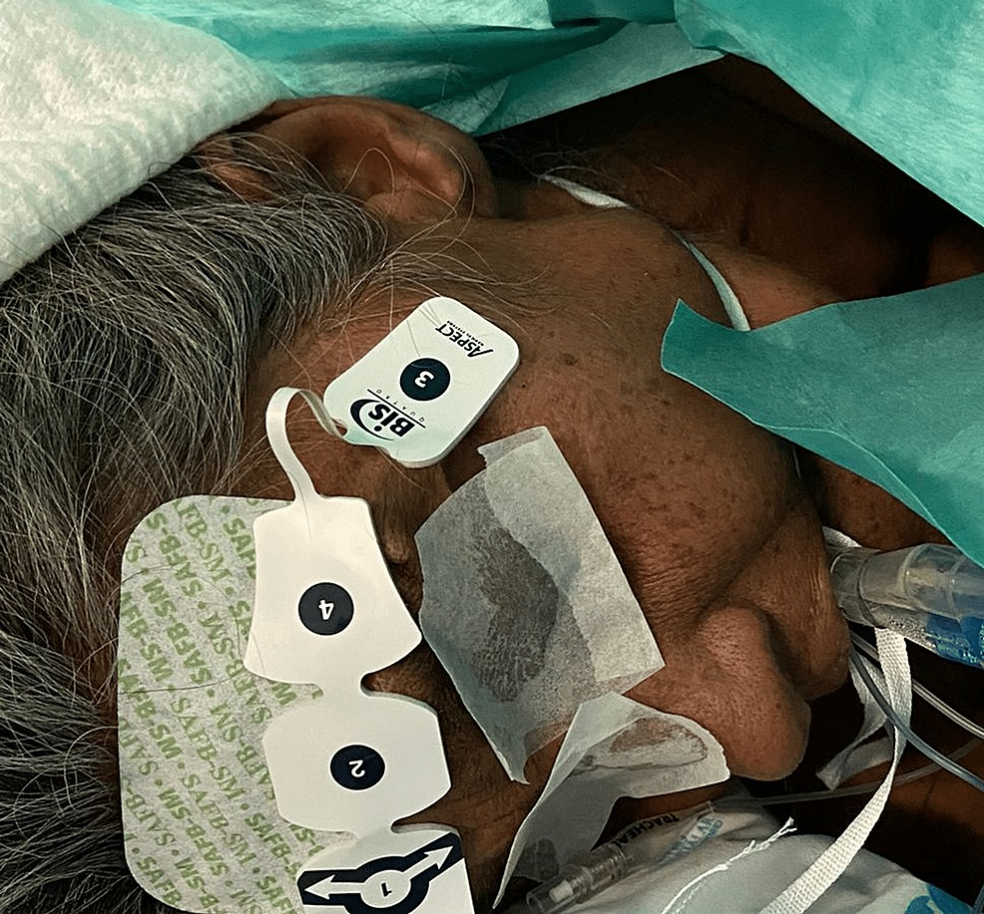
Caucasian patient with darker brownish skin color due to clofazimine-induced skin pigmentation

A 62-year-old Caucasian woman, with American Society of Anesthesiologists (ASA) physical status III, who had a history of chronic infection of bronchiectasis due to multidrug-resistant Mycobacterium abscessus, was scheduled for a right pneumectomy by video-assisted thoracoscopic surgery. She also had a past medical history of cerebrovascular disease, as she had experienced a TIA two years prior. Her usual medical therapy included montelukast, clofazimine, amikacin, acetylsalicylic acid, and inhaled glycopyrronium. After starting therapy with clofazimine, she had noticed a change in her skin color, which had turned to darker brownish (Figure 1).

Regarding preoperative laboratory values, she had a hemoglobin of 11.6 g/dl, while her coagulation studies, renal function, electrolytes, and arterial blood gas were normal. Pulmonary function tests showed a slight decrease in diffusing capacity for carbon monoxide, without other alterations. She also had a preoperative CT angiography of the cerebral arteries, which was normal.

The anesthetic plan consisted of general anesthesia, with standard ASA monitoring, invasive pressure monitoring, and cerebral oximetry monitoring using INVOS™ 5100C (Medtronic plc, Dublin, Ireland). Before administering any drug and while the patient was still alert, we monitored NIRS, adhering to the manufacturer’s instructions, and the values recorded were 15 on the left side and 26 on the right side, with one stable bar in the signal strength indication (SSI). After changing the adhesive pads, the values recorded were 15 on the left side and 26 on the right side. These values remained stable during the whole procedure.

We performed total intravenous anesthesia with target-controlled infusion (TCI) of propofol (effect-site concentration: 2-4 µg/ml) and remifentanil (effect-site concentration: 1-3 µg/ml). Neuromuscular block was maintained with rocuronium intermittent boluses. We performed orotracheal intubation with a left double-lumen tube, in which correct positioning was confirmed using fiberoscopy.

Total anesthesia time was four hours and no remarkable events were observed. There were two episodes of hypotension (median arterial pressure: <65 mmHg), which were promptly managed with phenylephrine bolus (100 µg) and lasted less than 10 minutes. One-lung ventilation was uneventful. We administered multimodal analgesia using intravenous (IV) paracetamol (1000 mg), IV tramadol (100 mg), and IV ketorolac (30 mg) and performed an ultrasound-guided erector spinae plane (ESP) block at the T7 level. After the surgery, the patient’s neurological status was unchanged and she was extubated. She was transferred to the surgical ICU for postoperative recovery. The postoperative period was uneventful and the patient was discharged home 10 days later.

## Discussion

Cerebral oximeters use NIR light to determine regional hemoglobin oxygen saturation (rSO_2_) in the frontal lobes. Two adhesive pads, applied over the frontal lobes, emit and capture reflected NIR light passing through the skin and cranial bone to and from the underlying cerebral tissue [7]. Baseline cerebral oximetry values should be obtained with the patient on spontaneous ventilation on room air and before the administration of any sedative drug. Normal values range from 60% to 80% [3]. Deviation in rSO_2_ from baseline level should be monitored and significant reductions in rSO_2_ levels may be identified and corrected, in order to maintain cerebral oxygenation. However, rSO_2_ values must not be interpreted in isolation since, as with all monitoring devices, cerebral oximetry has certain limitations, such as interference with blood from extracranial sources, electrosurgical equipment, sensor mispositioning, and NIR light transmission attenuation due to skin melanin [4,8,9]. Thus, all clinical information available and the patient’s physiological state must be considered when there are alterations in cerebral oximetry.

In our case, we decided to monitor cerebral oximetry due to our patient’s past medical history of TIA, and the fact that she was going to undergo a surgical procedure with expected hemodynamic and oxygenation-relevant events. When we monitored our patient before anesthetic induction, the rSO_2_ values were 15% on the left side and 26% on the right side. Since the patient was alert and with an unaltered neurological status at that time, we did not believe that these rSO_2_ values reflected our patient’s real cerebral oxygenation. SSI showed only one of four bars, which, according to the manufacturer’s manual, indicates a weak signal, but one strong enough to generate an accurate rSO_2_, with no further action required [10]. Nonetheless, we decided to change the adhesive pads, ensuring their proper placement. After switching sensors, the rSO_2_ values did not change and SSI was maintained with one stable bar.

Our patient had received treatment with clofazimine for chronic infection of bronchiectasis by a multidrug-resistant Mycobacterium abscessus, the prolonged use of which is associated with skin pigmentation. Clofazimine-induced skin pigmentation, its most common side effect, is believed to be caused by bioaccumulation and precipitation of the drug [6]. Skin pigmentation is an independent predictor of the level of rSO_2_, due to its effect of attenuating NIR light transmission, which alters cerebral oxygen saturation estimation [4]. Thus, our patient suffered from clofazimine-induced skin pigmentation, a condition that changed our patient’s skin color to a darker brownish tone, which may have interfered with the NIR light transmission, explaining the extremely low rSO_2_ values found. The relationship between high serum bilirubin or race and falsely lower rSO_2_ has been reported in the literature [4,5]. However, to our knowledge, there are no published cases describing the interference in cerebral oximetry by drug-induced skin pigmentation, a non-negligible limitation as 10-20% of cases of skin pigmentation are drug-induced [6].

Given the high risk of a perioperative stroke and the impossibility of monitoring cerebral oxygenation, we strictly followed the recommendations to reduce the risk of perioperative stroke [1] and implemented intraoperative strategies, such as the maintenance of a mean arterial pressure >70 mmHg and avoidance of hypocarbia.

## Conclusions

Cerebral oximetry should be interpreted in the context of all available clinical information since NIRS transmission can be influenced by several factors, and skin pigmentation is one of them. Besides race or high serum bilirubin concentration, other causes of skin color change must be considered, namely drug-induced skin pigmentation, as illustrated in the case we described. We hope that our findings will contribute to and encourage further investigation and refinement in the monitoring of cerebral oxygenation.
